# Towards a toolkit for global insect biodiversity monitoring

**DOI:** 10.1098/rstb.2023.0101

**Published:** 2024-06-24

**Authors:** Roel van Klink, Julie Koch Sheard, Toke T. Høye, Tomas Roslin, Leandro A. Do Nascimento, Silke Bauer

**Affiliations:** ^1^ German Centre for Integrative Biodiversity Research Halle-Jena-Leipzig, Puschstrasse 4, Leipzig 04103, Germany; ^2^ Department of Computer Science, Martin-Luther-University Halle-Wittenberg, Von-Seckendorff-Platz 1 06120 Halle, Germany; ^3^ Department of Ecosystem Services, Helmholtz-Centre for Environmental Research - UFZ, Permoserstr. 15, Leipzig 04318, Germany; ^4^ Friedrich Schiller University Jena, Institute of Biodiversity, Dornburger Straße 159, Jena 07743, Germany; ^5^ Department of Biology, Animal Ecology, University of Marburg, Karl-von-Frisch-Straße 8, Marburg 35043, Germany; ^6^ Department of Ecoscience, Aarhus University, C. F. Møllers Allé 8, Aarhus C 8000, Denmark; ^7^ Arctic Research Centre, Aarhus University, Ole Worms Allé 1, Aarhus C 8000, Denmark; ^8^ Department of Ecology, Swedish University of Agricultural Sciences (SLU), Ulls väg 18B, Uppsala 75651, Sweden; ^9^ Organismal and Evolutionary Biology Research Programme, Faculty of Biological and Environmental Sciences, FI-00014 University of Helsinki, Helsinki, Finland; ^10^ Science Department, biometrio.earth, Dr.-Schoenemann-Str. 38, Saarbrücken 66123 Deutschland, Germany; ^11^ Swiss Federal Research Institute WSL, Zürcherstrasse 111, Birmensdorf CH-8903, Switzerland; ^12^ Swiss Ornithological Institute, Seerose 1, Sempach 6204, Switzerland; ^13^ Institute for Biodiversity and Ecosystem Dynamics, Sciencepark 904, Amsterdam 1098 XH, The Netherlands; ^14^ Department of Environmental Systems Science, ETH Zürich, Universitätstrasse 16 Zürich 8092, Switzerland

**Keywords:** insects, arthropods, monitoring, ecosystem health

## Abstract

Insects are the most diverse group of animals on Earth, yet our knowledge of their diversity, ecology and population trends remains abysmally poor. Four major technological approaches are coming to fruition for use in insect monitoring and ecological research—molecular methods, computer vision, autonomous acoustic monitoring and radar-based remote sensing—each of which has seen major advances over the past years. Together, they have the potential to revolutionize insect ecology, and to make all-taxa, fine-grained insect monitoring feasible across the globe. So far, advances within and among technologies have largely taken place in isolation, and parallel efforts among projects have led to redundancy and a methodological sprawl; yet, given the commonalities in their goals and approaches, increased collaboration among projects and integration across technologies could provide unprecedented improvements in taxonomic and spatio-temporal resolution and coverage. This theme issue showcases recent developments and state-of-the-art applications of these technologies, and outlines the way forward regarding data processing, cost-effectiveness, meaningful trend analysis, technological integration and open data requirements. Together, these papers set the stage for the future of automated insect monitoring.

This article is part of the theme issue ‘Towards a toolkit for global insect biodiversity monitoring’.

## 1. Introduction to the theme issue

Insects are the most species-rich group of animals on Earth, and, together with other arthropods, make up most of the terrestrial animal biomass [1]. They provide a myriad of ecosystem functions and interact with species across the tree of life. They also provide services and disservices that make insects inextricably intertwined with human lives: for instance, many plants, including important agricultural crops, depend on insect pollinators [2]; other insect species are among our worst crop pests or are disease vectors for plants, animals and humans. For these reasons, we need a comprehensive toolkit for insect biodiversity monitoring and surveillance across the globe. This theme issue aims to make a major advance towards this goal.

Despite their importance, insects have long been under-represented in biodiversity monitoring—probably because of their limited appeal, and the challenges of accurate identification. Consequently, insect monitoring has largely remained limited to charismatic species, such as butterflies, to freshwater quality indicator species, and to species with obvious agricultural, silvicultural or medical importance. However, even so, the monitoring of these groups has mostly been restricted to a limited number of high-income countries. Other arthropod groups have only been monitored when and where academics or citizen scientists have had a personal interest and/or the required funding, leaving the majority of taxa and locations unstudied. Consequently, it has only recently been noticed that the abundance and, less frequently, the diversity of insects have significantly declined in many parts of the world [3–5], with formerly abundant species showing the strongest declines [6].

Traditionally, insect biodiversity monitoring (with the exception of butterflies and dragonflies) has required laborious morphological identification and counting of the collected individuals. This approach has several limitations: first, even for programmes with a limited taxonomic scope (e.g. one insect order or family), the workload of trapping and identifying thousands of individuals is enormous and unfeasible for most taxa. Secondly, the taxonomic expertise required for insect monitoring is rare or may not exist at all, especially at places where most species remain undescribed. These points are particularly relevant for all-taxa inventories. A vivid illustration of the limited throughput of such traditional monitoring approaches is the partial inventory of arthropods within 60 ha of tropical forest which took a decade to complete [7]. Similarly, a Swedish nation-wide inventory of arthropods took two decades to identify only approximately 1% of the material [8]. Third, the trapping and killing of insects increasingly raises concern among the wider public and citizen scientists [9], on whose volunteering efforts many monitoring programmes rely [10]. Hence, traditional methods are not a feasible option for large scale, all-taxa monitoring.

However, in order to meet the 2030 targets of the Convention of Biological Diversity [11], and to monitor progress on the United Nations decade of restoration and the sustainable development goals (SDGs; [12], in particular SDGs 12 and 15), the monitoring of insect biodiversity is crucial. For this, the use of lists of species' occurrences, and red lists are insufficient [13]. Instead, we need sustained, long term and standardized monitoring across the full spectrum of insect taxa and landscape types to capture their essential biodiversity variables (EBVs) [14]: species identity, population abundance, range, genetic diversity, phenology, body mass, species diversity and community composition, as well as ecosystem-level processes, such as species interactions and biomass flows. This will not only allow us to track changes in abundance and distribution of economically and medically important species, or select invasive and threatened species, but also for the many neglected insect groups.

Recently, technological developments have been made in various fields that hold enormous promise for insect monitoring and surveillance. Using these, it will soon be possible to automatically and autonomously detect and identify insects based on images, sounds, genetic traces and other properties, in many cases in a non-invasive way [15]. Such advances will transform insect monitoring from a labour-intensive to a computationally intensive field. These advances have already led to important insights into insect ecology and biodiversity (e.g. [16–18]), but with sustained development of the technologies, much more will be possible.

## 2. The contributions to four technological approaches in this theme issue

In this theme issue, we introduce the capabilities of four major approaches that have seen significant advances over the past decades and have reached various states of deployment readiness [15]—molecular methods, computer vision, autonomous acoustic monitoring and radar-based remote sensing. A number of reviews and case studies show the type of data and insights these approaches can provide, and demonstrate the range of ecological applications, while several perspective pieces discuss overarching themes, such as the possibilities for deployment across the globe.

We bring together international and multidisciplinary expertise addressing the aims of insect biodiversity assessments and monitoring. In total, 142 authors from 27 countries, with backgrounds as diverse as laser physics, genetics, taxonomy, ecology and computer science, have contributed their work. The contributions provide a broad spectrum of studies reflecting the state-of-the-art of the technologies, as well as challenges and opportunities for addressing monitoring questions from species inventories to community assemblages. The spatial and temporal scales that are covered in this theme issue range from local to continental and from snapshots to continuous recording. It is our intention that this theme issue is a resource for scientists, practitioners and policymakers interested in monitoring insects at various levels of scale and resolution.

Two other technological approaches to insect ecology and monitoring have only recently emerged and have not yet been applied extensively: photonic sensing (i.e. lidar) and ecotremology. Therefore, they could not be explicitly covered in this theme issue, but because of their potential, they are worth mentioning here. In photonic sensing, insects are detected as they pass through laser beams, providing rich information on their reflective properties, wing beat frequency, speed and direction [19]. In ecotremology [20], the vibrations produced by insects (and other animals) on plants and in the soil and other substrates can be detected with contact microphones [21] or lasers [22], providing information on the insect community producing these signals.

### (a) Molecular methods

Molecular methods identify the presence of species from their molecular traces (typically DNA or RNA), which can be extracted from collected insects [23], from a trapping medium (e.g. [24]), or from the wider environment (eDNA; [25–27]). The widespread use of such methods was kickstarted by the seminal contributions of Hebert *et al*. [23], who proposed that short pieces of standardized marker genes could serve as reliable keys to species identification. The rapid advance of sequencing techniques has revolutionized this field: sequencing platforms have become more efficient and more affordable, allowing not only the screening of large samples [28,29] but also efficient species discovery [30]. As a particularly exciting development, DNA barcodes can not only identify the species identity of insects themselves, but also reveal more of their Eltonian niches [31], including the plant species the pollen they carry belongs to [32], and their microbiomes, as shown by Łukasik & Kolasa [33]. However, the rapid development of molecular methods has resulted in methodological sprawl, raising pleas for a much-needed standardization (and at least comprehensive reporting) of methods. The adoption of general guidelines is clearly needed to make data comparable, and Iwaszkiewicz-Eggebrecht *et al.* [34] make an important contribution to make this happen. Another key challenge for current DNA-based methods is the reliable assignment of DNA-sequences to established taxonomy, which is crucial, since ecological information and traits are tied to taxonomic names. Although the vast majority of species have not yet been sequenced, the current reference libraries against which to match sequences are already immense (the global database BOLD hosting over 13M sequences), and will continue to grow. The vast amount of reference data puts a strain on the computational resources available for taxonomic assignment using these libraries. To assign a likely taxonomic name to a sequence, it is not only necessary to evaluate its similarity to all sequences in the library, but also to assess the (substantial) likelihood that the sequence is still missing from the library. Li *et al*. [35] make an important step in using graphics processing units for taxonomic assignment, scaling the tool to the task. With the data in hand, the downstream analysis of the data becomes a key priority. Again, the development of relevant methods has so far lagged behind the accumulation of data. Targeting the terrestrial and the aquatic realm, respectively, Li *et al*. [29] and Blackman *et al*. [28] demonstrate how the resulting data can be used for efficient landscape-level predictions of species distributions, community structure and the state of the environment. In an exciting outlook, Meier *et al*. [30] outline the path to efficient training of image recognition algorithms by drawing on DNA-sequenced specimens, thus combining two techniques outlined in this theme issue. Such integrated approaches can provide the route to high-throughput biodiversity discovery and monitoring.

### (b) Computer vision

Images capture information in a similar way to what humans observe with their eyes. Using computer vision, often in combination with deep learning of annotated images, images of organisms can be automatically identified, frequently to species or genus level. Together with the development of high-quality smartphone cameras, this has paved the way for the set-up of online portals for image-based species observations such as eButterfly, iNaturalist and ObsIdentify, which are aimed at citizen scientist involvement [36]. However, to make use of this technology for insect monitoring and biodiversity assessments, more standardized methods are needed.

In contrast to the other technologies covered in this theme issue, which are not size-sensitive and can capture all organisms within their sensors' ranges, specialized hardware is needed for the application of computer vision to insects and other small invertebrates. For instance, wildlife cameras are typically designed for recording vertebrates at several metres distance and usually cannot focus at the shorter distances needed for insect monitoring, or do not feature motion sensing technology that is triggered by small organisms. Recently, several camera trap designs have been developed to monitor insects at low maintenance costs (e.g. [37–39]). In parallel, imaging devices for laboratory studies of insect specimens are being developed that can efficiently capture rich information from bulk samples and single specimens [30,40,41]. Designing and assembling such custom-made systems made from cameras, mini-computers, a power source and additional memory, requires mechanical and electrical engineering skills [37,38], making the application of computer vision to insects an inherently interdisciplinary endeavour.

Since insect camera designs are still in their infancy, we ultimately need, but are a long way from, defining and implementing standards for hardware, data generation and image analysis. As a first step towards achieving this goal, Roy *et al.* [42] discuss metadata standards for hardware and image analysis. With the continued development of hardware and analytical algorithms, rigorous metadata recording will be fundamental for future trend analyses and, possibly, reprocessing of (historic) images [43].

### (c) Autonomous acoustic monitoring

Passive acoustic monitoring employs autonomous recording units (ARUs), which record the sounds of soniferous insects and other animals either continuously or intermittently (subsamples of minutes) in both the audible and ultrasonic spectrum [44]. Recorded sounds are usually automatically identified using artificial intelligence (AI) trained on annotated sounds from known species [45]. Newer and cheaper ARUs and better analytical methods, such as deep learning, have allowed for acoustic monitoring at unprecedented temporal and spatial scales [17], as well as the re-analysis of previously recorded sounds (i.e. bioacoustic time capsules [46]). Although ARUs have been embraced for automated biodiversity monitoring, only a minority (5%) of the 460 research articles on acoustic monitoring in terrestrial environments published between 1992 and 2018 targeted insects [47].

Autonomous acoustic monitoring can detect known natural and anthropogenic biodiversity gradients [48,49] and has proved to be a valuable tool for monitoring soniferous insects [17]. In this theme issue, two papers use passive acoustic monitoring to investigate the ecology of neotropical Orthoptera. Symes *et al*. [50] investigate the effects of moonlight, and Do Nascimento *et al*. [51] of habitat structure on their calling activity. Orthopterans are probably the best-studied group of soniferous insects globally, but the potential and taxonomic scope of acoustic monitoring has not nearly been fully tapped. Desjonquères *et al*. [52] provide a thorough review of the potential for underwater acoustic monitoring of insects, and Rodriguez *et al*. [53] develop acoustic monitoring for threatened pollinators.

To more fully tap this potential, global species monitoring needs enhanced libraries against which audio recordings of insects are compared, and the training of classifiers to recognize these sounds. Madhusudhana *et al*. [54] present one solution for this problem by developing a method for training a deep learning model using sparse data of poorly known species. Another option for expanding sets of available training data will be the sharing of annotated species- and group-specific insect recordings by scientists and citizens online.

### (d) Radar-based remote sensing

Radars emit electromagnetic beams that are reflected by objects in the air, and based on their echoes, these objects can be characterized. A variety of radar systems exists that differ in specifications, in the level of detail they provide on aerial objects and in spatio-temporal resolution [55]: small-scale radars can identify individual animals and characterize their body shape, size, wing beat frequency and other characteristics, but cover only relatively small aerial volumes [56]. By contrast, weather radar networks survey the atmosphere above areas stretching several hundreds to thousands of square kilometres but provide fewer and coarser characteristics of the biological targets detected [57].

It has been known for decades that radars can detect insects [58]. Particularly specialized small-scale entomological radars have provided fundamental insights into orientation, flight behaviour, migration patterns and biomass flows of high-flying insects [58]. However, as radars can only rarely identify flying insects to species level, this method has long been discarded from the monitoring toolbox. Yet, with their continuous operation and large-scale coverage of aerial habitats, radars can deliver information which complements that from other, finer grained, monitoring approaches.

Recently, both small-scale specialized and large-scale weather radars have seen exciting developments that expand the use of radars for insect monitoring: technical advances such as the upgrade of weather radars to dual-polarization devices and the development of new small-scale radars will yield more and/or better estimates of shape, size and variety of aerial objects, and thus, set the basis for improved classification. Parallel developments in algorithms for object classification, such as machine learning, will expand insect identification capabilities from radar signals. Finally, the roll-out of small-scale radars across larger regions and an improved access to high-quality data from continental weather radar networks means that monitoring can cover yet larger regions [59].

The contributions in this theme issue show-case several of these developments and demonstrate the potential of radars for contributing macroecological patterns to insect monitoring: First, Bauer *et al.* [55] review the use of radar for insect monitoring. Then, Drake *et al.* [60] develop biodiversity metrics that can complement the biomass and abundance measures from radar reflections, thereby enhancing the information that can be gained from radar monitoring. Gao *et al*. [61] relate insect abundances, flight altitude and orientation as derived from three small-scale radars to environmental factors, particularly, meteorological and celestial conditions. Haest *et al*. [62] use a European network of small-scale radars to exemplarily investigate diel activity patterns of insects. On a yet larger spatial scale, Tielens & Kelly [63] use the network of weather-radars in the contiguous USA to identify a latitudinal gradient in insect flight activity, and its variation across biomes.

## 3. Towards global insect biodiversity monitoring

**Figure 1.**
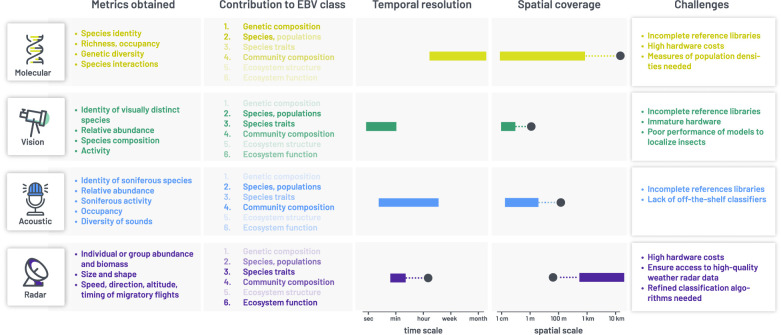
The complementarity and challenges for the widespread implementation of the four technological approaches to insect monitoring treated in this theme issue: molecular methods, computer vision, autonomous acoustic monitoring and radar-based remote sensing. The six classes of essential biodiversity variables (EBV's) to which each method contributes are shown with increasing opacity based on the assessed contribution of the technology (dark, strong contribution; light, little to no contribution). Temporal resolution refers to the duration of a typical sample, i.e. the aggregation of information to form one sample (minutes between photos, minutes of acoustic recording or radar sweeps, or the days to millennia over which molecular traces aggregate). Spatial coverage indicates the spatial area one sample can cover. For temporal resolution and spatial coverage, the typical range of application is depicted as a solid bar, with the extreme limits of the technology depicted as a black circle. The final column summarizes the most important challenges for widespread implementation; additionally all technologies require specialized skills and training and all face the challenge of handling and processing large data-volumes.

The technologies covered in this theme issue each have their unique, yet highly complementary, set of strengths and limitations, spatial and temporal scales, and biodiversity metrics they can provide (figure 1). Combining them would make insect biodiversity assessments, monitoring and surveillance more comprehensive, potentially include the majority of insect species, and cover unprecedented spatial, temporal and taxonomic scales. Such integrated monitoring is within reach but a number of challenges remain to be tackled, some of which reflect the various stages of deployment readiness, while others concern cross-technology integration.

First, the technologies covered here share a reliance on advanced devices, computational power and infrastructure. Consequently, they have predominantly been developed, tested, and used in high-income countries in the temperate zone, even though biodiversity is highest in the tropics. This disparity needs to be levelled, lest the currently existing bias in biodiversity data availability [64] widen even more. Two perspective pieces deal with this issue. Sanchez *et al.* [65] emphasize the needs of biomonitoring using modern technologies in the Global South, while Brydegaard *et al.* [66] present some frugal solutions to meet these needs. As the lack of biodiversity data often results from lack of resources, the sharing of knowledge and devices and a move away from centralized, resource-intensive platforms towards distributed, frugal solutions could contribute to local capacity building and alleviate some of the global inequalities [66].

Second, there is a strong need for definition, implementation and adoption of methodological and data-sharing standards. This is especially pertinent for technologies that are still undergoing rapid development in software or hardware (molecular methods, acoustics and vision). For each of these technologies, a profusion of approaches has recently been developed, but methodological reporting has not kept pace. Hence, even data derived using the same technology is often not comparable, and difficult to integrate with data from other approaches. Therefore, setting standards for methods, meta-data and archiving within one technology is a prime need and should consider the requirements for integration with other technologies. Iwaszkiewicz-Eggebrecht *et al*. [34] and Roy *et al*. [42] make important steps towards establishing standards for molecular monitoring and computer vision, respectively. There is also a need for harmonized storage of data and metadata. Svenningsen & Schigel [67] outline the possibilities for the archiving of machine-derived data on the global biodiversity information facility. Still, even when methodological standards have been agreed upon, the continued development of each of the technologies poses a real challenge for the standardized quantification of EBV's, especially over long time periods. Van Klink [43] proposes strategies to ensure the usefulness of the collected data for future trend calculations.

Third, the currently available reference libraries of DNA sequences, images and sounds needed for classification are patchy at best, and will need to be expanded to incorporate a meaningful part of global biodiversity. This is not just a matter of recording the properties and DNA sequences of species, but must take into account the variations of, e.g. sounds, wing-beat frequencies or coloration with temperature, season, or age of the insect. This variability in properties will also need to be accounted for by collecting reference data under various environmental conditions, and/or using sophisticated modelling approaches. Given that most insect species are rarely observed, new methods are required to include these species despite the scarcity of records (see [54]). To accommodate the massive growth of these libraries, improved matching algorithms are needed [29].

A shared challenge across all technologies is the need for very specific and advanced (bio)informatics skills, and the computational resources to store and process large data volumes. This requires constant training of people to stay up-to-date with the latest developments, and may lead to competition with other economic sectors for skilled workers.

## 4. A grand vision

Ultimately, we envision an integrated insect monitoring programme that is deployed for many years across different habitats, landscapes, countries and continents, covering a diversity of strata and insect taxa to close global gaps in insect monitoring. One critical component of such a programme will be simultaneous installations of several or all technologies (multi-sensor stations [68,69]) at the same locations, interspersed by smaller stations with small-scale radars, camera traps and/or audio recorders. This will allow the coarse but large-scale information on insect biomass distribution and flight activity from weather radar networks to be used as a basis for modelling biodiversity in unmonitored places in the landscape based on detailed local information.

A critical step towards such an integrated monitoring system is a high level of automation that explicitly aims at reducing human labour and maintenance. Autonomously operating devices are already available for radar and acoustic monitoring, while insect camera traps with AI identification are still under development, for example using insect's attraction to light (e.g. [37]), or coloured screens [39]. For molecular methods, some steps can be automated, e.g. using automatic bottle changers for Malaise traps [68] and parts of the laboratory protocols [30], but human labour will probably continue to be needed for parts of field and laboratory work.

Naturally, such a general purpose insect monitoring programme could be complemented with data from already deployed specialized automated monitoring devices, e.g. targeted at agricultural pests [70–72] or invasive species [73]. Likewise, since the devices not only detect insects and other invertebrates, the information on vertebrates and micrometeorological conditions [74,75] could feed into their respective monitoring programmes. Ultimately, the data recorded by all devices should be automatically transferred, processed and archived, so that the derived EBVs are available at everyone's fingertips in near-real time.

Other important complementary information may come from existing monitoring schemes, which are often supported or run by volunteers. Data from these programmes have been, and will continue to remain, invaluable. Extant time series offer a bridge between the past, current and future state of the biosphere, and can, in most cases, not easily be switched to new methods without losing the compatibility between past and future data. Continuing these efforts will be essential to enable hindcasting of past biodiversity and its change.

As these programmes often rely on volunteers, they are an important way nature enthusiasts come into direct contact with insects in their natural habitats. To keep volunteers engaged, Sheard *et al.* [36] review the need to consider the potential consequences of technological developments also for citizen scientists and peoples' connection with nature: the use of novel technology in citizen science projects can increase the ecological detail recorded and, importantly, reduce the taxonomic expertise required for participation. However, this may reduce the inclusivity of such programmes, e.g. owing to higher financial costs of participation, technological expertise needed, and the reliance on digital infrastructure, and thus, jeopardize widespread implementation [36].

Still, even with an integrated, cross-technology monitoring system at hand, some inherent features of the natural world need to be acknowledged: (i) species with cryptic lifestyles, specific habitat needs or poor mobility will be difficult to detect (for example, subterranean and cave-dwelling species, specialists of remote and extreme habitats, and species able to avoid trapping); and (ii), even among the detectable species, a significant number are rare and thus, infrequently detected [13,76] despite great sampling efforts (in the Swedish nationwide survey with 200 sites, 13% of species were only observed once and only at a single site [13]). Consequently, these rare and hard-to-detect species will not be adequately monitored, nor their trends observed and quantified in EBVs. For those, we will need other approaches, possibly involving labour-intensive campaigns targeted at specific biomes, habitats, or lifestyles.

## 5. Conclusion

With a comprehensive automated insect monitoring system that integrates information from traditional and specialized monitoring programmes, we will increase our knowledge of insect species trends and distributions and close gaps in global biodiversity data. This will provide the basis for informed conservation and management actions and effective policies to halt (or reverse) the rapid loss of biodiversity, as well as benefit society through the development of automated warning systems for agricultural and silvicultural pest outbreaks, the spread of vector borne diseases, and invasive species.

## Data Availability

This article has no additional data.
